# CT-Derived 3D Printing for Coronary Artery Cannulation Simulator Design Manufacturing

**DOI:** 10.3390/bioengineering9080338

**Published:** 2022-07-25

**Authors:** Helvina Vika Etami, Rochmi Isnaini Rismawanti, Vita Arfiana Nur Hanifah, Herianto Herianto, Yarabisa Yanuar, Djoko Kuswanto, Dyah Wulan Anggrahini, Putrika Prastuti Ratna Gharini

**Affiliations:** 1 Department of Cardiology and Vascular Medicine, Faculty of Medicine, Public Health, and Nursing, Universitas Gadjah Mada, Yogyakarta 55281, Indonesia; vikaetami@gmail.com (H.V.E.); wulan.anggrahini@ugm.ac.id (D.W.A.); 2 Dr. Sardjito General Hospital, Yogyakarta 55281, Indonesia; 3 Faculty of Medicine, Public Health, and Nursing, Universitas Gadjah Mada, Yogyakarta 55281, Indonesia; rochmi.isnaini.r@mail.ugm.ac.id (R.I.R.); vita.arfiana.nf@mail.ugm.ac.id (V.A.N.H.); 4 Center of Additive Manufacture and System, Faculty of Engineering, Universitas Gadjah Mada, Yogyakarta 55281, Indonesia; herianto@ugm.ac.id (H.H.); yarabisay@gmail.com (Y.Y.); 5 Department of Industrial Design, Faculty of Creative Design and Digital Business, Sepuluh Nopember Institute of Technology, Surabaya 60111, Indonesia; crewol@prodes.its.ac.id

**Keywords:** 3D printing, coronary intervention, education, simulation, simulator

## Abstract

Mastering coronary angiography requires practice. Cadavers and animals do not accurately represent the human anatomical body, and practicing with actual patients has medical safety issues. Simulation offers safe and realistic conditions for cardiology intervention training. In this study, we propose a novel 3D printed simulator that contains physically realistic anatomy and has four access points. It increases safety for patients and students, and production is low-cost. We aimed to make and validate this simulator design as a prototype for coronary cannulation training. It was designed using computed tomography (CT) scan data of aorta, coronary, and heart models, and was printed by 3D printing with resin materials consisting of 75% or 85% clear resin and 25% or 15% flexible resin additive. The simulator was constructed with a camera above the simulator with a degree of LAO of 30°/0°, a display table, and an acrylic box. Twelve validators were interviewed for their expert opinions and analyzed by a qualitative method. They scored the simulator’s suitability on a four-point Likert scale questionnaire. They described the simulator as having admirable values for all aspects (85.8%), curriculum suitability (92%), educational importance (94%), accuracy (83%), efficiency (78%), safety (87.5%), endurance (81.2%), aesthetics (80.7%), storage (85.4%), and affordability (85.8%).

## 1. Introduction

Percutaneous endovascular interventions with minimally invasive procedures are rapidly increasing because of their improved benefits, decreased hospitalization, and minimal use of anesthetic drugs [1]. The operators have to hone their skills, which require practicing in order to augment the actual procedural performances [2]. Learning by using cadavers is expensive and difficult to maintain, while animal use is not representative of the human body [3]. Furthermore, procedures performed on patients without practice may do harm to the patients, and the issues surrounding the current awareness of the potential for human error in medical practice still remain a hot issue regarding patient safety [4].

Simulation is used for high-risk and precise procedures; it is suitable for interventional cardiology learning and can improve the students’ acquisition of new skills [5]. This benefit is more prominent for amateurs than for experts [6]. Ideally, the skills of surgeons who have trained with simulators are better than those who have little or no training [7]. Furthermore, students’ interests and abilities will increase with new teaching media, such as the use of simulators. Training in coronary cannulation techniques is a basic skill for cardiology residents. The use of three-dimensional printing is now well-known and increasing in medical settings for learning, surgical preparations, bioprosthetic production, and organ-assisted device-making [8,9,10].

This angiography procedure may cause complications to patients, such as dissection of the aorta or coronary vessels, arrhythmia, myocardial infarction, bleeding, or death. Simulation allows students to practice without risk of these complications [11]. The 3D printing may be used to make interventional cardiology simulators that have complex structural and high precision models and fast production time. The 3D printing data come from computerized tomography (CT) scans, magnetic resonance imaging (MRI), single-photon emission tomography (SPECT), or 3D trans-thoracic echocardiography (TTE)/trans-oesophageal echocardiography (TOE) [9,12,13]. Printing materials depend on the teaching purpose. For example, acrylonitrile butadiene styrene (ABS) and polylactic acid (PLA) with fused deposition modelling (FDM) can print hard materials for bone structures. Binder jetting (BJ) and stereolithography (SLA) are often used for liquid materials such as resin to make cartilage, tumors, liver segments, and heart parts. Polyjet (PJ) is applied to the combination of materials and can be used to print rubber with rigid photopolymers for arteries, mitral valves for catheter interventions, and arterial clips for cerebral aneurysms [14].

Simulator production must have replication precision that will replicate the real setting environment, available tools, and student mentality. In the learning process, students are given feedback from the instructor [15]. Normal CT scan data from coronary arteries to femoral arteries from some patients are segmented, edited with reduced noise, then smoothened, thickened, and stabilized to make the simulator design before simulator printing and construction [16]. The quality of the image is dependent on the properties of the medical imaging used and the type of printing method, and the cost spent is reflected in the image size and selection, materials used, and printing methods [17]. Resin is widely used from industrial sources because it has good strength and adhesion, is chemically resistant and stable, has low shrinkage, and is easy to apply. The cross-linked density of the resin determines the transparency and flexibility [18].

Virtual reality (VR) simulators show the topology of the 3D prototype; the images do not describe a concrete structural depth and physical organ model compared to 2D images, and they do not require high-cost maintenance [5,19]. This research aimed to produce a simulator for coronary artery cannulation with all puncture sites that can serve as a concrete structural and physical organ model. It mimics aortic characteristics by material selection and enables students to observe anatomical and functional aspects for procedural learning purposes. Resin can also be used for 3D printing materials in the simulator production, which can significantly lower costs [16].

The contributions of this study area are described below:

- Students are able to learn the coronary artery cannulation with four access points, while seeing and feeling the real physical anatomy of the organ.
- The 3D printing technology gives an advanced and cost-effective medical simulator with simple, readily-available materials for manufacturing.
- The selection of 3D printing materials for the simulator production resembles the proposed organ and can be adjusted for the procedure learning purposes, providing a clear and flexible 3D printout.

## 2. Materials and Methods

This was a research and development study to develop and assess the validity of a new product. The subjects were chosen based on their expertise related to the simulator function [20]. The design was built and printed with 3D technology into a prototype, and then prototype validation was conducted by qualitative methods and a questionnaire. This research was conducted in the Dr. Sardjito General Hospital, Yogyakarta, Indonesia; Faculty of Medicine, Public Health and Nursing, Universitas Gadjah Mada; Integrated Digital Design (iDIG) Surabaya Technology Institute; and Execute 3D Printing Workshop Yogyakarta. The total of 12 study subjects included experts in anatomy (n = 2), engineers (n = 2), and an interventional cardiology team, which consisted of interventional cardiologists (n = 2), an interventional fellow of cardiologist (n = 1), interventional nurses (n = 3), and radiographers (n = 2). They participated in the research from January 2021 to February 2022. Exclusion criteria included: (1) did not meet the criteria of the cardiology interventional team, and (2) did not give informed consent.

### 2.1. Three-Dimensional Prototype Design

**Figure 1:**
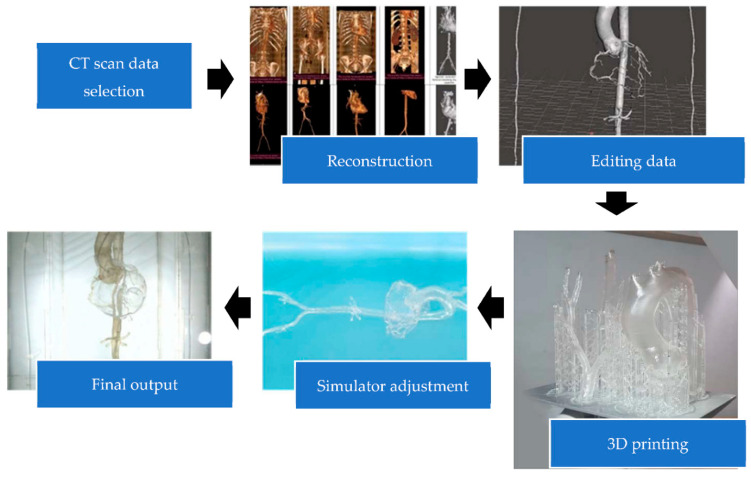
Manufacturing process of the simulator.

The simulator, which consisted of the coronary arteries, aorta and the branches, right and left iliac artery, right and left femoral artery, subclavian artery, brachial artery, and radial artery, was designed from anatomically normal CT scan data from IntelliSpace Phillips version 10.1 (Philips, Best, The Netherlands). The CT scan data were chosen and modified based on the imaging cardiologist, interventional cardiologist, and anatomy experts to modify the organ structure for coronary artery cannulation importance. Then, the data were edited by Autodesk Meshmixer 3.5 (Autodesk Inc., San Francisco, CA, USA) and Blender 2.93 (Blender Foundation, Amsterdam, The Netherlands) for organ segmentation and smoothening of the design surface and saved into an. STL format. Data were transferred into G-code before printing. The best material, the method for printing, printout thickness, and simulator construction were discussed by both cardiologists and engineer experts. In this step, the lattice structure (the support to strengthen the 3D printout) was also printed and had to be removed manually. The assembly of the 3D printing was completed in an acrylic box and display table to make the simulator (Figure 1). A LAO 30°/0° view was set from the camera holder to resemble the real procedure.

### 2.2. Three-Dimensional Design and Prototype Validation

There were two steps for the validation. First, the soft file data were examined by anatomists and an interventional cardiologist to evaluate the suitability of the anatomical design structure. Next, the second validation step was performed by anatomists, engineering experts, and an interventional cardiology team (interventional cardiologists, a fellow of interventional cardiology, interventional nurses, and radiographers) after formation of the simulator prototype. In this step, interviews were conducted with all of the subjects according to their expertise. The interview guide was based on their realm of expertise. A 4-point Likert scale on the anatomical structure of the 3D printout was scored by the anatomy experts and interventional cardiology team. The questionnaire that related to simulator suitability for learning importance was completed by the interventional cardiology team. The subjects’ responses were collected until the data reached redundancy, when there was nothing new offered from the expert opinions concerning the simulator.

### 2.3. Analysis

The analysis was conducted using qualitative methods for the interview analysis. The interview data, which were saved in audio recording or video, were summarized into open coding, based on the important or main parts of the interviews that contained new expert opinions. Data were made into short narrations or connections to simplify the research understanding. If the data display was strong, then summaries of the simulator’s suitability were built. The questionnaire on the prototype suitability of the anatomical 3D printout was calculated as a percentage based on scoring of the suitability of each anatomical structure by the anatomy experts and interventional cardiologist. The data on the simulator suitability for learning from the questionnaire filled out by interventional cardiology team were calculated manually by Sugiyono’s formula to determine the suitability percentage (Table 1) [20]. Tables and graphics were included to emphasize the results.

$$\mathrm{Suitability}=\frac{\mathrm{Obtained}\;\mathrm{score}}{\mathrm{Criteria}\;\mathrm{score}}\times 100\%$$

Obtained score = Sum of all aspects and subject score

Criteria score = highest criteria × number of items × number of subjects

### 2.4. Ethical Issues

This research was conducted with approval from the Medical and Health Research Ethics Committee of the Faculty of Medicine, Public Health and Nursing, Universitas Gadjah Mada, Yogyakarta, Indonesia with the number KE/FK/0926/EC/2021. All of the subjects were explained the research procedures and signed the informed consent form before participating.

## 3. Results

### 3.1. Simulator Model Making

The researchers made non-systematic observations of the process of coronary angiography learning in Dr. Sardjito General Hospital, Yogyakarta, Indonesia, where they used the traditional method of observation, explanation, and training by the trainer, identified as the interventional cardiologist. There were five CT scans from 21 patients used to design the coronary arteries, aorta and the branches, iliac artery, femoral artery, subclavian artery, and radial artery from IntelliSpace Philips version 10.1. The data were edited by Autodesk Meshmixer 3.5 (Autodesk Inc., San Francisco, CA, USA) and Blender 2.93 (Blender Foundation, Amsterdam, The Netherlands) to delete, construct, and smooth the design (Figure 1). Anatomical design validation included opportunities for the anatomists and interventional cardiologists to ask questions about the suitability of the anatomy and the simulator design performance.

### 3.2. Design Validation

The design was representative of the anatomy. However, design correction from both validators was needed. The common iliac artery and femoral artery branches were maintained to resemble the catheter road and mark the false route pitfall, and some non-existent parts of both artery branches were removed. The abdominal aorta branches, which consisted of superior and inferior mesenteric artery, coeliac trunk, and renal arteries, were exposed to mark the aorta location and the thoracic aorta branches. The left common carotid artery and left subclavian artery were more medially located than normal anatomy. Therefore, shifting both arteries to the left part of the thoracic aorta was required. The superior extremity artery was reviewed to add the ulnar artery for puncture procedural importance, but the CT scan data had a limitation to the contrast coloring of the ulnar artery so adding the ulnar artery to this design could not be pursued. There was no comment on coronary arteries (Figure S1, Supplementary Material). In addition, the replenishment of clearly distinguishable markers, such as the skeleton, was expressed by the anatomist to determine the catheter position. An acrylic box was applied to protect the 3D printout and hold the water; the laminar flow was required to be similar to blood flow.

### 3.3. Design Printing

An exploration of raw materials for the 3D print of the design was conducted to find those that had transparent, flexible, and strong characteristics. The design was also printed hollow so as to be anatomically normal. This was also considered to be transparent so that a catheter was able to be visualized as it crossed the hollowed aorta. The printout needed to be flexible and strong to resemble the aorta characteristics, and so did the repetition function of the simulator for skills training. A combination of clear and flexible resin with a consistency of 75% or 85% of clear resin and 25% or 15% of flexible resin additive was the most suitable mixture for the 3D printing of the design (Figure S2 of the Supplementary Material). The mixture of 100% clear resin or 90% clear resin with 90% flexible resin additive produced a whitish blur that was unclear in the visualization. However, the 50–50% mixture was known to have low tear durability. The sticky printout was produced by the higher percentage of flexible resin and is known to hamper catheter and wire movement. All of these combinations were printed by the stereolithography (SLA) 3D printing technique, which allowed fluid material for 3D printing. Coating with polyurethane (PU) was also applied to the printed design to strengthen the model’s exterior and make it clear. The heart design used the vacuum forming method with 3 mm mica, which compressed the 3D printout from polylactic acid (PLA) material by the fused deposition modeling (FDM) technique.

The mixture of latex had a very good elasticity, but this method was not good enough because the color did not look transparent. This technique was suitable for making an artery or vein puncture manikin. However, the molding technique with a silicone mold, which was made by 3D-printed poly-lactic acid (PLA), was not effective and required a long time to create. Gravitational forces also affected the resin mold, so the surface would not be the same as others. Additional data are shown in Figure S3 of the Supplementary Material.

### 3.4. Simulator Forming

**Figure 2:**
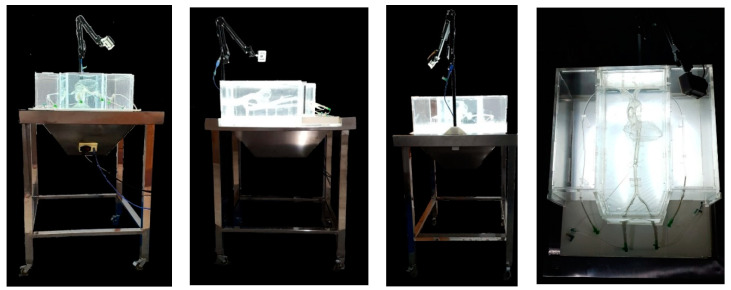
Three-dimensional printed coronary artery cannulation simulator.

The 3D printing had a lattice structure that supported the printout, and this material had to be removed manually. Then, the 3D printout design was placed in a large transparent acrylic box in order to give protection and 40 L of water placement to lubricate the hollowed model. A 850 cm stainless-steel display table, with four 4-volt light-emitting diodes (LED) lamps and a 220-volt electricity cable inside, was utilized to position the model and acrylic box. Four wheels were placed under each table’s legs to ease simulator mobilization. The camera holder was attached to the display table on the top of the acrylic box. Meanwhile, the 1080 pixel (full HD) camera was placed above the 3D model with the position of LAO 30°/0°, which became known as the coronary cannulation view (Figure 2). The simulator’s total dimension was 60 × 60 × 120 cm with a weight of 35 kg. This dimension was more efficient than that of a real patient setting. The picture from the camera was transferred into the monitor by a USB cable, so the trainers and students could perform the actions of the procedure while looking at the monitor.

### 3.5. Prototype Validation

A total of 12 validators (2 anatomy experts, 2 engineering experts, 2 interventional cardiologists, 1 interventional fellow cardiologist, 3 interventional nurses, and 2 interventional radiographers) were asked for their expert opinions about the simulator prototype. The main simulator criteria were the educational importance and curriculum suitability, accuracy, efficiency, safety, endurance, aesthetics, storage, price, and all of the performances. This simulator was valued as completely feasible by all validators, with an average percentage of 85.8%. The aim of learning coronary artery cannulation was given a percentage value of educational importance from all validators of 93.7%, a value of enriching students’ competence in coronary artery canulation of 96.8%. This simulator had a completely feasible value for curriculum suitability (92%), with the concept of the curriculum (93.7%) and object clarity (90.6%).

**Figure 3:**
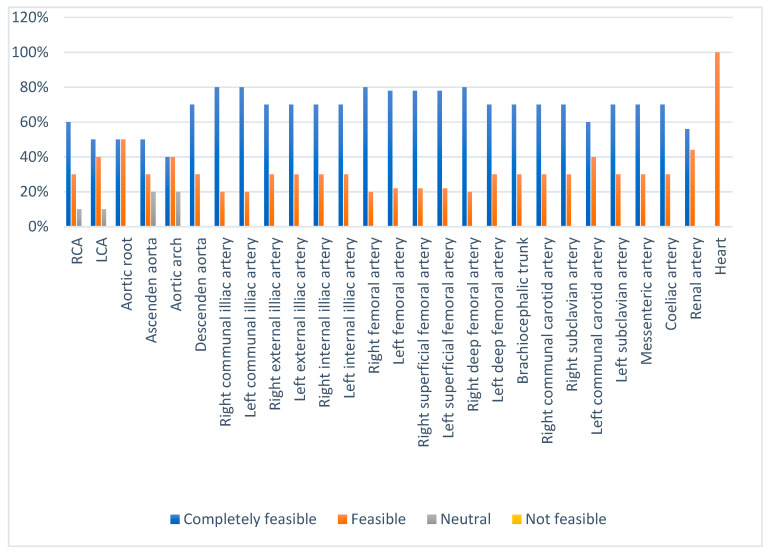
Three-dimensional printout of the anatomical suitability from the validator assessment.

**Figure 4:**
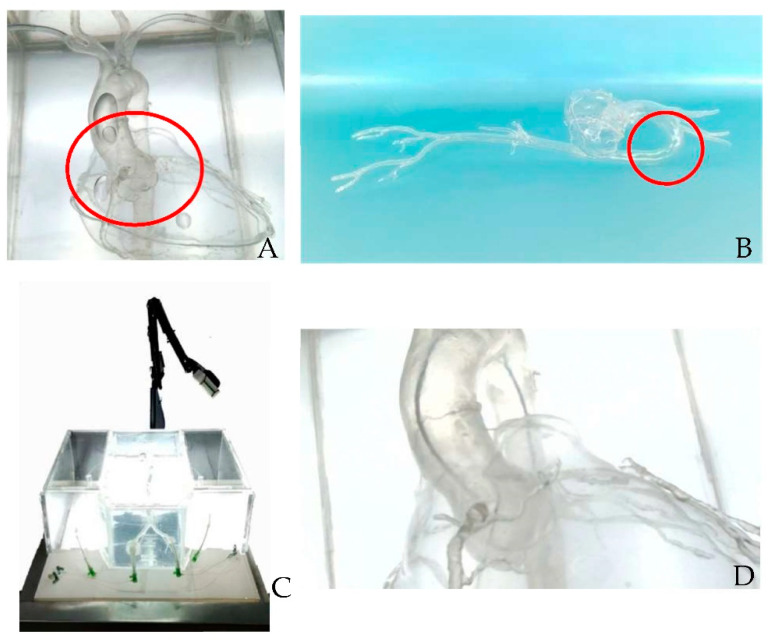
(**A**) Evaluated area to reduce the thickness. (**B**) Evaluated area of aortic arch angulation. (**C**) LAO 30°/0° camera position. (**D**) The view of LAO 30°/0°.

This simulator was valued as very accurate (83%) with anatomical coherence (88.7%) when examined by anatomy experts and the interventional cardiology team, which was further valued for both a positional component (79.2%) and printout coherence (85.4%). We used real patient CT scan data for the design and provided a feasible printout (Figure 3) (additional data regarding the 3D printout anatomical suitability scoring percentage is presented in Table S1 of Supplementary Materials). The inserting and manipulating of the wire and catheter were completely feasible (87.5% and 84.3%, respectively) for either clockwise or counterclockwise catheter manipulation. However, the insertion and manipulation of the wire and catheter were hard to perform (81.2% and 75%, respectively). The difficulty of the catheter insertion was caused by the material used. Additionally, the 3D material and the aortic arch angulation might interfere with the wire and catheter movement. The catheter manipulation was found to be hard with the JL catheter because of the angulation of the distal aortic arch (Figure 4B). However, the total efficiency of the simulator was valued as feasible with a percentage of 79.2% on average by the validators.

The aortic root clarity had to be increased by reducing the thickness of the junction between the coronary artery and aorta and the number of heart model attachments on the aorta (Figure 4A). The view position should be LAO 30°/0° in respect to the real position of coronary artery cannulation by adding a marker bow to fix the position (Figure 4C,D). Indicators for catheter engagement must be supplied by adding color injection to the coronary artery. The material used was valued at 79% by the average of all validators. Moreover, this simulator was easy to assemble (78%), which was achieved only by adding water to the acrylic box and plugging the cable into electricity and the USB to the monitor. Hand support was added by making a table extension to support the operator’s hand. An automatic table for up and down table movement was needed to match the operator’s height. Table S2 of the Supplementary Materials lists the evaluation score of the coronary artery cannulation simulator by the cardiology intervention team.

The safety of this simulator was completely feasible (87.5%), considering the material used (87.5%) and utilization (87.5%). Electricity was a concern due to water use above electricity. Therefore, the connection of the acrylic box had to be tight with an interlocking connection, and the wire and catheter access had to be fixed to prevent leakage (Figure S4, Supplementary Materials). Valve addition in the access might help with leakage prevention and provide wire and catheter insertion and removal. Direct current (DC) electricity was advised by the engineering expert since AC has a higher current level and continuous distribution, which can easily lead to an electrical injury. Adding support to femoral access should be effective as a fixator to reduce movement. The shape was elegant, but some improvement was needed for better aesthetics (valued at 80.7%). The acrylic connection and simulator cover was placed tidily, as shown in Figure S4 of the Supplementary Materials, including the replacement of wireless gadgets. The simulator dimension is smaller than the real procedure and easy to maintain (85.4%). A hand or tight manikin should be added to increase aesthetics and provide students with simulated cannulation insertion access.

The endurance of the simulator was valued as 81.2% by all validators, with 82.3% for strength and 80.2% for ease of maintenance. The simulator strength was tested by moving the simulator 1.2 km on a steep road without water added to the acrylic box, but if the water in the acrylic box was full, it would easily spill out from the box. Therefore, empty or half-filled water is recommended for simulator movement. The problems with the material used were color changes due to ultraviolet (UV) and water exposure. We found that the resin printout color changed to yellow because of UV exposure and whitish blur due to water exposure. These were concerns because the simulator used water and LED lights, which included UV light. The experiment used LED lights for 14 days, which did not alter the resin printout color. However, exposure to water changed the resin printout color to white, and the changes were apparent in 5 days with exposure to well water and 14 days with tap water. Additional data are shown in Figure S5 of the Supplementary Materials.

These color changes need further in vitro investigation for long-term changes and environmental factors and simulator maintenance. Furthermore, mass testing is needed for the further improvement of the simulator. Materials that are more suitable for UV and water exposure need to be investigated. Simulator storage was easy (85.4%), and the simulator needed a dark cover to prevent UV exposure and a way to empty the water after simulator usage to prevent color changes. The addition of a wheel at each table foot made it easy to move. The simulator cost a total of IDR 17.000.000,00 (USD 1170), which consisted of aortic 3D printing (IDR 7,000,000.00/USD 481), a display table (IDR 3,000,000.00/USD 206), an acrylic box (IDR 5,000,000.00/USD 344), electronics (IDR 1,500,000.00/USD 103), and coronary artery cannulation tools (IDR 500,000.00/USD 35), but these costs did not include non-material fees. Table S3 of the Supplementary Material summarizes the opinions and feedback of the coronary artery cannulation simulator from the validators.

## 4. Discussion

This simulator concept was achieved by considering the cardiology student requirements to increase their coronary cannulation skills as described for concept suitability from the curriculum, since the ability to perform examinations and construct a clinical interpretation of invasive diagnostic tests of coronary artery disease that starts from puncture to coronary artery cannulation is one of the cardiologist’s competencies that requires repetitive training [22]. This study provided a simulator for coronary artery cannulation learning that was easy to use, safe, and cost effective from CT scan data. The curriculum suitability was reflected by the concept suitability of the curriculum and object clarity for coronary artery cannulation. Stereolithography printing allowed fluid material printing, which was utilized in resin and produced a high-resolution printout that afforded object clarity.

Active learning has a better outcome than passive learning and simulating the real experience will help students to remember 70–90% of the learning because students will enjoy studying more [23]. Significant technical skills development has been achieved by amateur students with simulator training [7,24]. The simulator was made based on the students’ intellectual ability. Students’ motivation and interest in coronary artery cannulation learning will increase with the presence of simulators as learning media. Hence, students’ competence in coronary artery cannulation will increase.

The component accuracy of this simulator was created to imitate real patients and situations, which also altered simulator efficiency. Segmenting the coronary artery, aorta and the branches, and peripheral extremity branches consumed time and required multidiscipline experts from cardiology imaging, cardiology intervention, and the anatomy department. Therefore, the simulator dimensions must be arranged according to real human anatomy from CT scan data in order to ease wire and catheter insertion and movement. However, the disadvantage of flexible material was that it was easy to deform, which changed the angulation angle of the aorta (Figure 4B) and hindered wire and catheter movement.

The material selection that resembled blood vessel elasticity character and provided clear transparent visualization was challenging. Urethane acrylate oligomer, methacrylate oligomer, resin, and polyurethane were utilized to build an artery design for surgical training, which requires elastic materials to mimic the vessel [25]. Coronary artery cannulation is best positioned in LAO 50° because the aortic root, coronary artery, and sinus of Valsalva do not overlap [26]. This positioning was referred to as the camera position by adjusting the camera holder. A contrast agent was injected into the coronary artery to visualize the coronary artery during the angiography procedure [27]. A whole hollowed coronary artery must be formed to allow fluid color flow, which was still difficult to print due to the thin layered and fragile 3D-printed coronary artery. In addition, an investigation of non-lasting color for the resin is needed to maintain printout clarity. Learning the best resolution and visibility of specific anatomical interests and data imaging interpretation will require proper attention and sufficient time [28].

Simulator endurance was determined after testing. The strength was tested by the distance traveled by this simulator. The color changes were explained by the resin’s character. Resin is a photo-initiator that is hardened by UV (250–450 nm) exposure. Polyurethane, the material that was used for coating, turned yellow after UV exposure [29]. This was explained by the oxidation reaction of the polymer backbone. Irradiation modifies the physical and chemical characteristics of polyurethane surfaces and causes color changes and degradation. In addition, urethane bridges the quinone–imide oxidation structure and becomes a chromophore of yellow color [30]. The sun is a source of full-spectrum UV radiation with a various wavelength that consists of UV-C (100–280 nm), UV-B (280–315 nm), and UV-A (315–400 nm). A UV LED lamp has a narrower spectral output, which emits 365 nm, 385 nm, or 405 nm wavelengths [31]. To protect from photodegradation, UV absorbers or stabilizers are used against UV light with miscellaneous mechanisms. Inorganic UV absorbers spread light, while converting UV light to heat, higher wavelengths, and radical interceptors, and free radicals are captured by organic UV absorbers [32]. Thus, the UV absorber and a dark cover are needed to protect the simulator to avoid color changes. For proper storage, the simulator should be placed away from sun exposure.

The color change differences caused by the water sources might be affected because of the different biological properties between the well water and tap water. The more the resin is exposed to water, the more water is stuck into the resin and causes resin softening due to degradation, which also affects the resin and causes color changes [33]. Polyurethane was used as a coating to strengthen the printout, but this was sensitive to humidity because of the free NCO group and had poor storage stability. Polyurethane, modified by glycidyl, strengthens the epoxy layer and makes a flexible cross-linking reaction [34]. These color changes can be alleviated by reducing water exposure by emptying the water after simulator usage.

Simulation technology has been used by some industries, including the medical field, for high-risk situational training [6]. The coronary artery cannulation procedure uses X-ray, in which the radiation can affect the students’ and patients’ safety, and can also cause complications that impact the patient [35,36]. Alternating current (AC) has a higher current level than DC, and the distribution requires synchronization and continuous change. Voltage changes will effectively pass into excitable tissues and skin, which leads to electric shocks when the current is high [37,38,39]. Therefore, DC was recommended by the engineer experts to replace AC in this simulator due to safety issues.

Virtual computerized media, such as CathlabVR from CAE Healthcare, Angio Mentor from Simbionix, SimSuite from Medical Simulation Corporation, and Procedicus VIST from Mentice, also provide cardiology interventional learning that includes coronary artery cannulation in coronary angiography procedures with 3D pictures that are observed via the monitor. They use single access from the femoral artery, or alternatively, Procedicus VIST uses dual access from femoral and radial arteries [5]. There are simulators that also use 3D printing technology but have different purposes. A specific normal heart and pulmonary artery were made by only focusing on the anatomy learning and surgical decisions [25,39]. The mitral stenosis heart model was constructed by 3D printing for percutaneous mitral annuloplasty learning [40]. A three-dimensional aortic model was used for endovascular training with or without fluoroscopy [16,41]. In comparison to those virtual media, this 3D printing anatomy printout of the simulator can be easily visualized in a real-size model on the operation table directly. This also brings the benefits of showing the basic anatomy and triggering students’ intellectual ability to imagine the anatomical position of the coronary angiography in addition to the skill training of coronary artery cannulation. Four access points from the right and left radial and femoral arteries with a fully constructed simulator enriches the student’s insights and skills for coronary artery cannulation procedure.

One of the limitations of 3D printing is the inability to translate organ flexibility and movement [14]. However, blood pulsation can be mimicked with the combination of other computational technologies. Radial pulse waveforms can be produced by a robotic tonometry system. This technology might be used to learn arterial puncture and diagnosis techniques [42]. A 3D blood flow model has been developed with the computational simulation by considering blood as a non-Newtonian fluid [43].

A small number of the validators might interfere with the validation result. A 3D-printed model could be tested for both cardiology students and angiography trainees to explore greater educational validation and simulator usage. Large mass production and usage are needed to investigate long-term endurance. Small print dimensions will further result in a longer production time. All of the limitations may be addressed in future studies. Overall, the 3D printing of coronary artery cannulation simulation models has the opportunity to improve coronary cannulation skills and may benefit both patient and student safety.

## 5. Conclusions

This study produced a 3D-printed coronary artery cannulation model for simulation and training purposes. We successfully developed a clear and flexible simulator with highly specific organ images and characteristics in a real setting procedure by CT scan data. Feedback using Likert scales provided evidence of its suitability as a prototype for a 3D-printed medical model, although some improvements were needed to upgrade the simulator. The printed model has specifications of a 3D-printed coronary artery cannulation model from CT scan data, light from LED lamps, a video circuit, an acrylic box, and a display table. This simulator provides educational value of diagnostic and coronary artery cannulation skills, while proving efficient, realistically accurate for wire and catheter manipulation, and aesthetic in shape and color, having good endurance, less technical maintenance, and safe materials, being free of radiation and fully portable, and having a relatively low cost. Hollowed coronary arteries, a 3D-printed model for artery and vein puncture, and artificial laminar blood flow are needed for further research.

## Figures and Tables

**Table 1:** Percentage of suitability [21].

Score	Category
<40%	Not feasible
40–55%	Neutral
56–75%	Feasible
76–100%	Completely feasible

## Data Availability

The data presented in this study are available upon request from the corresponding author.

## Supplementary Materials

The following supporting information can be downloaded at: https://www.mdpi.com/article/10.3390/bioengineering9080338/s1, Figure S1: Simulator design from CT scan data; Figure S2: The mixture of clear resin and flexible resin additive; Figure S3: Materials and production trials for simulator forming; Figure S4: Evaluated area for aesthetic, endurance, and safety; Figure S5: Color changes are caused by UV and water exposure; Table S1: Three-dimensional printout anatomical suitability scoring percentage; Table S2: Evaluation score of coronary artery cannulation simulator from the cardiology intervention team; Table S3: Opinion and feedback on the coronary artery cannulation simulator from the validators.
