# Supplementary material for: CT-Derived 3D Printing for Coronary Artery Cannulation Simulator Design Manufacturing
Source: Bioengineering (Basel). 2022 Jul 25;9(8):338. doi: 10.3390/bioengineering9080338 (PMC9330152; doi:10.3390/bioengineering9080338)
Supplement: Supplementary file 1 [file bioengineering-09-00338-s001.zip › bioengineering-1795395-supplementary.pdf]

Supplementary Materials

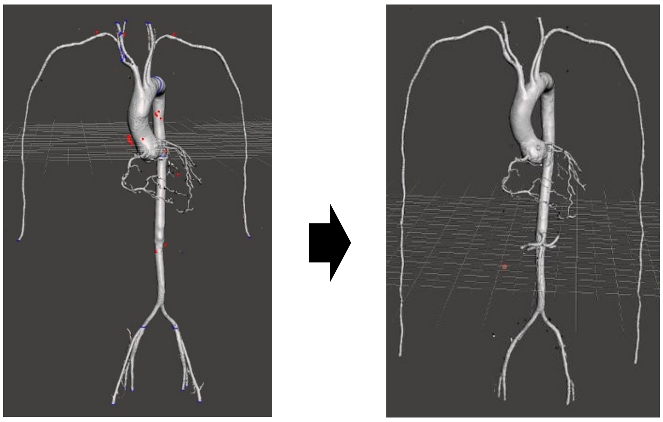

Figure S1. Simulator design from CT scan data. First design (left) and evaluated design (right) for coronary artery cannulation simulator by validators

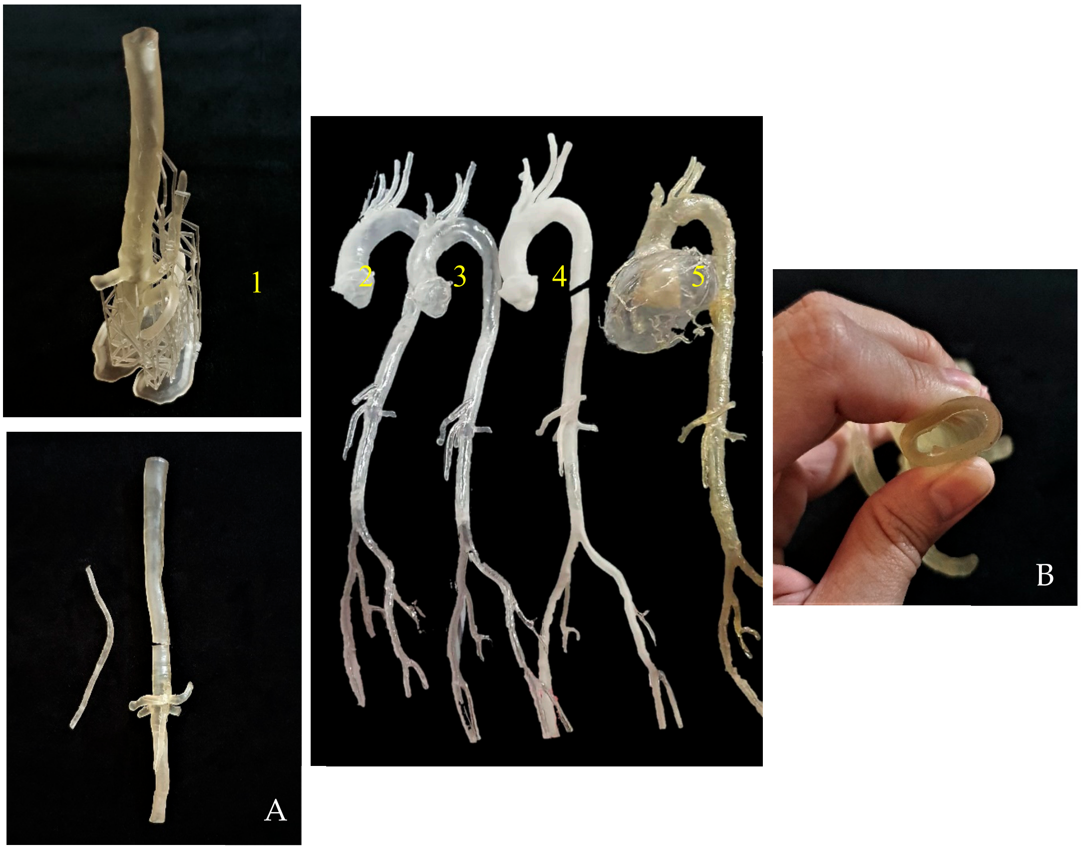

Figure S2. The mixture of clear resin and flexible resin additive. (A) Mixture of 90% clear resin and 90% flexible resin additive (1), 75% clear resin and 25% flexible resin additive (2), 85% clear resin and 15% flexible resin additive (3), 100% clear resin (4), and 50% clear resin and 50% flexible resin additive (5). (B). The flexibility characteristic of the 3D printout.

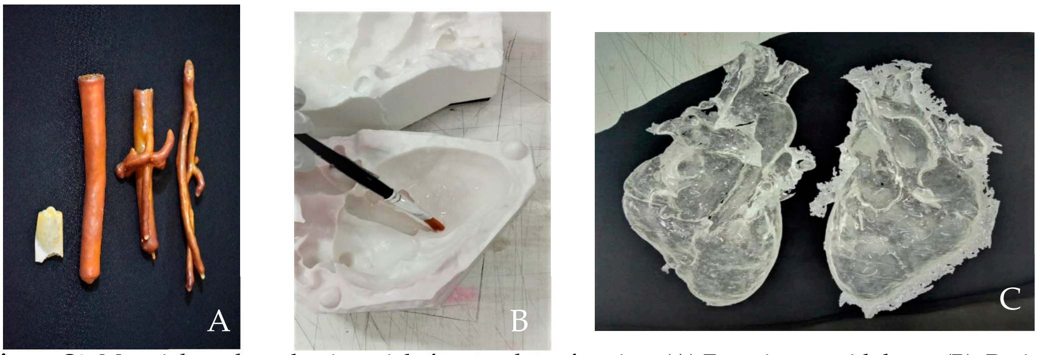

Figure S3. Materials and production trials for simulator forming. (A) Experiment with latex. (B). Resin administration to silicone mold. (C). Resin mold from silicone mold.

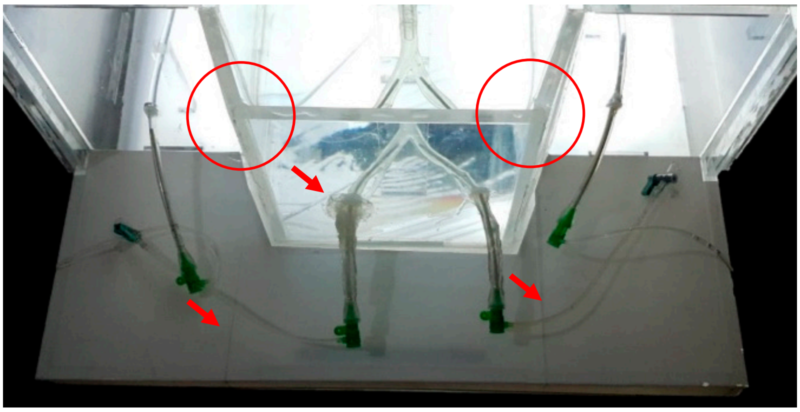

Figure S4. Evaluated area for aestethic, endurance, and safety. Evaluated area to tidy (red arrow) and strengthen the acrylic connection (red circle) to prevent leakage

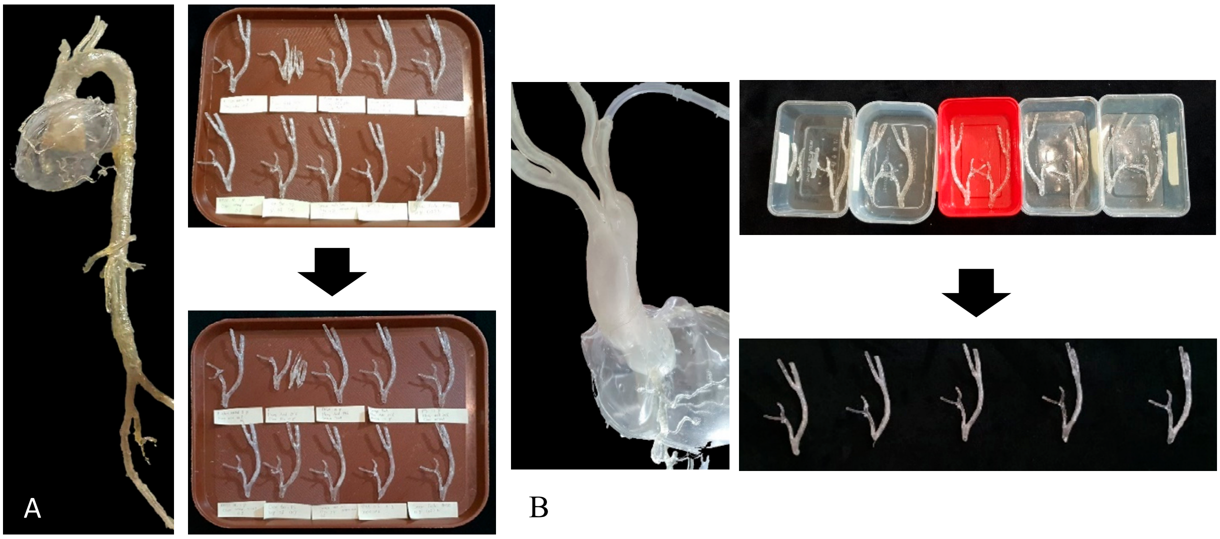

Figure S5. Color changes are caused by UV and water exposure. (A) Color change due to UV exposure (left) and experiment of LED lamp exposure for 14 days showed no color changes (right). (B) Color change due to well water exposure for 5 days (left), before experiment of tap water exposure (right top), and color change after 14 days of tap water exposure (right bottom)

Table S1. 3D printout anatomical suitability scoring percentage

No	Valued Criteria (Anatomy)	Completely Feasible		Feasible		Neutral		Not Feasible		Total
		N	Percentage	N	Percentage	N	Percentage	N	Percentage	
1	RCA	6	60%	3	30%	1	10%	0	0	10
2	LCA	5	50%	4	40%	1	10%	0	0	10
3	Aortic root	5	50%	5	50%	0	0	0	0	10
4	Ascenden aorta	5	50%	3	30%	2	20%	0	0	10
5	Aortic arch	4	40%	4	40%	2	20%	0	0	10
6	Descenden aorta	7	70%	3	30%	0	0	0	0	10
7	Right communal illiac artery	8	80%	2	20%	0	0	0	0	10
8	Left communal iliac artery	8	80%	2	20%	0	0	0	0	10
9	Right external illiac artery	7	70%	3	30%	0	0	0	0	10
10	Left external illiac artery	7	70%	3	30%	0	0	0	0	10
11	Right internal illiac artery	7	70%	3	30%	0	0	0	0	10
12	Left internal iliac artery	7	70%	3	30%	0	0	0	0	10
13	Right femoral artery	8	80%	2	20%	0	0	0	0	10
14	Left femoral artery	7	78%	2	22%	0	0	0	0	9
15	Right superficial femoral artery	7	78%	2	22%	0	0	0	0	9
16	Left superficial femoral artery	7	78%	2	22%	0	0	0	0	9
17	Right deep femoral artery	8	80%	2	20%	0	0	0	0	10
18	Left deep femoral artery	7	70%	3	30%	0	0	0	0	10
19	Brachiocephalic trunk	7	70%	3	30%	0	0	0	0	10
20	Right communal carotid artery	7	70%	3	30%	0	0	0	0	10
21	Right subclavian artery	7	70%	3	30%	0	0	0	0	10
22	Left communal carotid artery	6	60%	4	40%	0	0	0	0	10
23	Left subclavian artery	7	70%	3	30%	0	0	0	0	10
24	Messenteric artery	7	70%	3	30%	0	0	0	0	10
25	Coeliac artery	7	70%	3	30%	0	0	0	0	10
26	Renal artery	5	56%	4	44%	0	0	0	0	9
27	Heart	0	-	10	100%	0	0	0	0	10
**Total**		**173**	**65%**	**87**	**33%**	**6**	**2%**	**0**	**0**	**266**

Table S2. Evaluation score of coronary artery cannulation simulator from cardiology intervention team

Valued Aspect	Valued Criteria	Obtained Score	Criteria score/ Maximal	∑	I	F	P	R
						(%)		
Curriculum suitability	Concept suitability from curriculum	30	32	93.7	87.5	100	100	87.5
	Object clarity for coronary artery cannulation	29	32	90.6	75	100	100	87.5
	**Subtotal**	**59**	**64**	**92**	**81.2**	**100**	**100**	**87.5**
Educational importance	Suitability to student intellectual ability	30	32	93.7	100	100	100	75
	Improve student competence of coronary artery cannulation (before real procedure)	31	32	96.8	100	100	100	87.5
	Increase student motivation and interest	29	32	91.6	87.5	100	91.7	87.5
	**Subtotal**	**90**	**96**	**94**	**95.8**	**100**	**97.2**	**83.3**
Efficiency	Easy to use							
	- Insert *wire* and catheter	27	32	81.2	75	75	100	75
	- Manipulate catheter and *guidewire*	25	32	75	62.5	75	100	62.5
	Easy to assemble	24	28	78	87.5	75	100	50
	**Subtotal**	**76**	**92**	**78**	**75**	**75**	**100**	**62.5**
Accuracy	Component position accuracy to real patient	26	32	79.2	87.5	75	91.7	62.5
	Picture production	27	32	85.4	75	100	91.7	75
	Insert *guidewire* and catheter	28	32	87.5	75	100	100	75
	Manipulate *guidewire* and catheter	27	32	84.3	75	100	100	62.5
	Material used	26	32	79	75	75	91.7	75
	**Subtotal**	**134**	**160**	**83**	**77.5**	**90**	**95**	**70**
Aesthetic	Shape and size to study room	24	28	84.4	87.5	75	100	75
	Color	25	32	77	75	75	83.3	75
	**Subtotal**	**49**	**60**	**80.7**	**81.2**	**75**	**91.6**	**75**
Endurance	Strong	26	32	82.3	87.5	75	91.7	75
	Easy to maintain	26	32	80.2	87.5	75	83.3	75
	**Subtotal**	**52**	**64**	**81.2**	**87.5**	**75**	**87.5**	**75**
Safety	Material used	29	32	87.5	100	75	100	75
	Utilization	29	32	87.5	100	75	100	75
	**Subtotal**	**58**	**64**	**87.5**	**100**	**75**	**100**	**75**
Storage	Storage	28	32	85.4	100	75	91.7	75
	Mobility	28	32	85.4	100	75	91.7	75
	**Subtotal**	**56**	**64**	**85.4**	**100**	**75**	**91.7**	**75**
Price	Suitability to facility and institution (*feasibility*)	26	28	93.7	100	100	100	75

Suitability of learning benefits (*cost-effectiveness*)	26	28	93.7	100	100	100	75
**Subtotal**	**52**	**56**	**93.7**	**100**	**100**	**100**	**75**
**Total**	**626**	**720**	**85.8**	**87**	**85.8**	**96**	**74.4**

Notes :Σ = average of all subjects, I = interventional cardiologist, F = interventional cardiologist fellow, P = interventional nurse, R = interventional radiographer

Table S3. Opinion and feedback of coronary artery cannulation simulator from validators

I. Curriculum suitability
Engineer, 41 years old : "About my first impression of this simulator is very excited, I can't imagine how important is the simulator function, the importance is we can't practice direct to the patient and probandus because of safety, so I think this simulator is amazing."
II. Educational Importance
Cardiology fellow, 33 years old : "This is good for residents to learn with this simulator before applying to real patients, especially for the first week when the students are not allowed to do the procedure. They can learn the basic manipulation for coronary artery cannulation first from this simulator"
III. Efficiency
Interventionist, 69 years old: "This one should be evaluated, this angulation one…maybe this aortic arch is more angulated so that it is influencing the catheter manipulation."
IV. Accuracy
Interventionist, 39 years old: "The coronary cannulation uses LAO position, the most important aspect is for the true cannulation position and you have to make it flexible whether the position of the camera or the manikin position. The simulator's purpose is also for replacing learning media for cardiology residents before implementing the real procedure. So, they can imagine how the LAO or RAO is."
V. Aesthetic
Interventional nurse, 51 years old : "You can replace it with hand models, so it is like a true procedure. And you can add support, can be acrylic or soft support at femoral access to reduce leakage risk."
VI. Endurance
Anatomy expert, 40 years old : "You must know about the endurance. If this simulator is last for a long time, the buyer will think, about which part need to be fast replaced. So, this can be considered for the turnover model."<br><br>Engineer, 43 years old : These inventions about color changes and another lack ness can be used for simulator maintenance. Improvement for the simulator is needed for the next version."
VII. Safety
Engineer, 43 years old : "The safety concern is the electricity. DC electricity is preferred to AC in order to prevent electrical accidents. This acrylic connection must be tight, you can use a locked model."

Interventionist, 65 years old : " This catheter and wire access must be more fixed. It is better if you give like a valve in this access so the catheter and wire are easy to insert and remove like the real procedure."
VIII. Storage
Cardiology fellow, 33 years old : "This simulator is not too big, it will be saved easily in educational room."
IX. Price
Radiographer, 52 years old : "The price is not expensive compare to the function. I can't imagine how important it is."
